# Vitamin K and Hallmarks of Ageing: Focus on Diet and Gut Microbiome

**DOI:** 10.3390/nu15122727

**Published:** 2023-06-12

**Authors:** Lu Dai, Denise Mafra, Paul G. Shiels, Tilman M. Hackeng, Peter Stenvinkel, Leon J. Schurgers

**Affiliations:** 1 Aging Research Center, Department of Neurobiology, Care Sciences and Society, Karolinska Institutet, 17165 Stockholm, Sweden; lu.dai@ki.se; 2 Department of Clinical Science, Division of Renal Medicine, Intervention and Technology, Karolinska Institutet, 17165 Stockholm, Sweden; 3 Graduate Program in Nutrition Sciences, Fluminense Federal University, Niterói 24020-141, RJ, Brazil; 4 Wolfson Wohl Translational Research Centre, Institute of Cancer Sciences, University of Glasgow, Bearsden, Glasgow G12 8QQ, UK; 5 Department of Biochemistry, Cardiovascular Research Institute Maastricht (CARIM), Maastricht University, 6211 LK Maastricht, The Netherlands; 6 Institute of Experimental Medicine and Systems Biology, RWTH Aachen University, 52056 Aachen, Germany

**Keywords:** vitamin K, ageing, gut microbiome, food pattern

## Abstract

Vitamin K and vitamin K-dependent proteins have been reported to be associated with a large spectrum of age-related diseases. While most of these associations have been deduced from observational studies, solid evidence for the direct impact of vitamin K on cellular senescence remains to be proven. As vitamin K status reflects the complexity of interactions between dietary intake, gut microbiome activity and health, we will demonstrate the pivotal role of the diet-microbiome-health axis in human ageing and exemplify how vitamin K is implicated therein. We propose that food quality (i.e., food pattern) should be highlighted beyond the quantity of total vitamin K intake. Instead of focusing on a single nutrient, exploring a healthy diet containing vitamin K may be more strategic. As such, healthy eating patterns can be used to make dietary recommendations for the public. Emerging evidence suggests that dietary vitamin K is a modulator of the diet-microbiome-health axis, and this needs to be incorporated into the investigation of the impact of vitamin K on gut microbial composition and metabolic activities, along with host health outcomes. In addition, we highlight several critical caveats that need to be acknowledged regarding the interplay between diet, vitamin K, gut microbiome and host health that is pivotal for elucidating the role of vitamin K in ageing and responding to the urgent call of healthy eating concerning public health.

## 1. Introduction

There has been an unprecedented increase in the number and proportion of people aged >60 years old worldwide. This will accelerate in the coming decades, particularly in developing countries. In 2020, for the first time in history, people aged >60 years outnumbered children < 5 years. By 2050, this global aged population will have more than doubled to 2.1 billion, outnumbering adolescents and young people aged 15–24 years [1]. As there is little evidence that older people today are in better health than previous generations [2], this demographic change will tremendously impact health care. Health span (years of healthy living) still lags behind the increase in life span (years of living) achieved over the past century, and this deficit may be increasing in some countries (https://www.nrscotland.gov.uk/news/2022/scots-spending-more-lifetime-in-poor-health, accessed on 3 February 2022). According to WHO (2021), healthy ageing is “The process of developing and maintaining the functional ability that enables well-being in older age” [3].

Consequently, the global ageing population is becoming overwhelmed by age-related diseases and multi-morbidities, such as chronic kidney disease (CKD), cardiovascular disease (CVD), type 2 diabetes, obesity, cancer and neurodegenerative disorders [4]. Furthermore, health in older age is not equally distributed around the planet but varies regionally.

Many age-related chronic diseases and physiological dysfunctions related to the burden of lifestyle can be prevented and even reversed by implementing healthy lifestyle interventions [5,6]. It is worth noting that food is a single and strong determinant for optimizing human health. The Global Burden of Diseases, Injuries, and Risk Factors Study (GBD) 2019 [7] has reported that the global population was exposed to 47% adverse health risks from an unhealthy diet (e.g., a diet low in vegetables and fruits, whole grains and high in red/processed meat, sugar, and sodium), ranking as the second and third leading risk for total attributable death in both females and males (13.5% and 14.6% of total death, respectively). Moreover, as recently advocated by the EAT-Lancet Commission [8], a substantial shift from an industrialized to a healthy plant-based diet requires immediate action, as it would be expected to avert about 11 million deaths annually. With such a large magnitude of effect attributed to the link between diet and health, it is rational to highlight healthy eating as a direct preventive and therapeutic strategy to combat ageing and age-related diseases. Another potentially strong underpinning factor in the relationship between diet and health is the gut microbiome, which has emerged as a contributory factor to age-related health [9]. Given the key role of diet in determining the structure of function of the gut microbiome [10], the crosstalk between diet, the gut microbiome and host health have the potential to provide a holistic strategy to promote healthy ageing and reduce the gap between health span and life span, underpinned by a solid scientific evidence base. Pertinent to this is vitamin K, among many other nutraceuticals and bioactive compounds, which have gained prominence over the past decade in research on ageing [11]. Vitamin K status is a complex reflection of the milieu of dietary intake and utilization, gut microbiome composition and health conditions. This review will demonstrate the pivotal role of the diet-gut microbiome-health axis in ageing and age-related disease and exemplify how vitamin K is implemented in such a scenario to promote healthy ageing.

## 2. Vitamin K, Diet, and Healthy Ageing

### 2.1. Healthy Diet and Ageing

An unhealthy diet, characterized by a suboptimal intake of fruit, vegetables, and whole grains and high consumption of processed food products high in saturated fat, with added sugar and salt, constitutes a significant risk factor for poor health [12]. The shift towards a more carnivorous diet in modern Western society over the last century has resulted in a diet-associated “diseasome of ageing” [13]. A key hallmark of an industrialized diet is the intake of processed food, which not only causes excessive calorie intake [14] but also induces intestinal barrier permeability and microvascular disease [15]. As the consumption of processed red meat, in contrast to unprocessed red meat, has been linked to an increased risk of incident dementia [16], it is plausible that processed food per se is a culpable driver of the burden of lifestyle disease. The association between diet quality and age-related chronic disease has been well demonstrated [17,18,19], highlighting the potential interaction between overall dietary patterns and human health. Recent results from two large prospective cohorts combined with a meta-analysis of 26 prospective cohort studies have indicated that a high intake of fruit and vegetables was associated with reduced total and cause-specific mortality and that the lowest risk was associated with an intake of five servings per day of fruit and vegetable, thus providing succinct evidence and a recommendation for healthy eating [20]. Among many other nutrients, vitamin K1 intake links the consumption of vegetables/fruit and associated health benefits [21,22]. The benefits of healthy eating have also been supported by the Health and Retirement Study (HRS) and the Health Care and Nutrition Study (HCNS), where dietary quality was shown to be an effective means to reduce the progression of mobility limitations among elderly adults, as well as to modify the effect of multimorbidity on progressive disablement [23]. For vitamin K, dietary intake of foods rich in K1 or K2 has shown a promising role in combatting Alzheimer’s disease and cognitive decline in older adults [24,25]. Thus, it is encouraging that adherence to food quality can be implemented as a modifiable factor to improve well-being and promote healthy ageing in older populations. Indeed, this thesis of such a salutogenic diet can be used as a modern interpretation of the Hippocratic “food as medicine” concept, recently highlighted as a promising strategy to combat CKD and other age-related diseases [26,27].

### 2.2. Vitamin K Source, Metabolism, Recycling, and Its Role in Ageing

**Figure 1:**
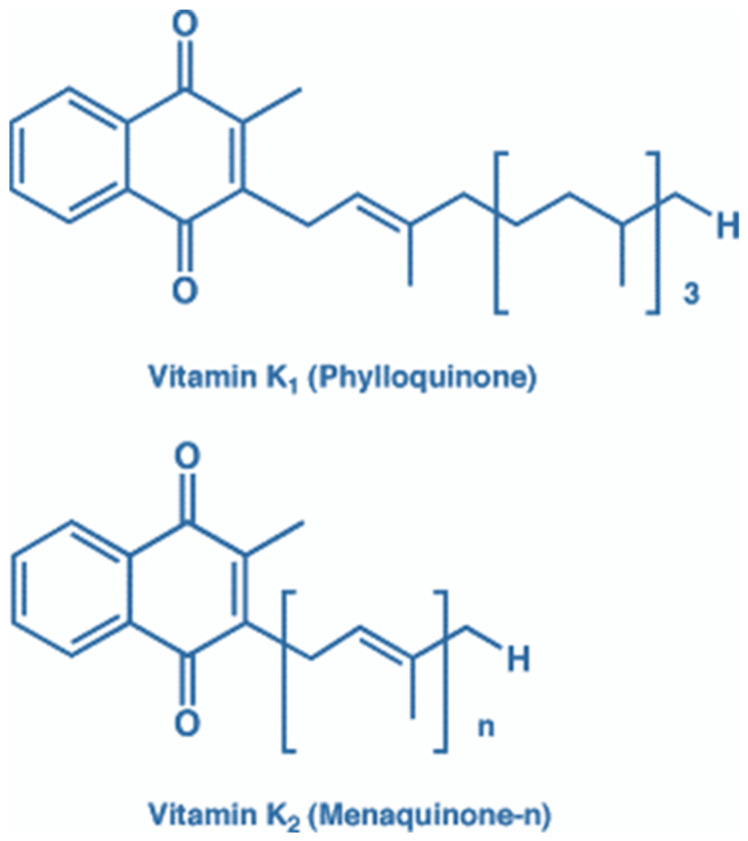
Vitamin K comprises a common naphthoquinone ring structure required for binding to the gamma-glutamyl carboxylase enzyme. The side chain of vitamin K derivatives is different. Vitamin K1 (phylloquinone) has a side chain comprising four isoprene units, with one unsaturated unit. Vitamin K2 (menaquinones, MKs) contains a range of K2 vitamins with a variety of isoprene units (ranging from 1 to 14) at the 3-position of the quinone structure, which is all unsaturated.

Natural forms of vitamin K comprise different naphthoquinone derivatives with various long aliphatic side chains. There are two significant forms comprising vitamin K1 (phylloquinone) and vitamin K2 species (menaquinones; MKs) (Figure 1). There is a broad variation in phylloquinone content from different food sources, including green leafy vegetables, such as collards, spinach, and kale, or vegetables such as turnip and broccoli and some fruits, such as dried prunes, kiwi and avocado, vegetable oils, and cereal products [28,29]. The bioavailability of phylloquinone from these foods has yet to be determined but is likely to vary, being least bioavailable in green vegetables (where phylloquinone is tightly bound to chloroplast membranes) and most bioavailable in processed food. Dairy products (e.g., yoghurt and cheese) and low-temperature stored/fermented vegetables (e.g., sauerkraut) are the major food source of vitamin K2 [30,31,32]. However, synthesis by colonic microflora (through the shikimate pathway) likely contributes to systemic levels. Lactic acid bacteria, for example, used in food manufacturing to ferment and preserve dairy products and vegetables, can synthesize vitamin K2 (MK5-MK9). In the Western diet, dairy products are the most consumed group of fermented products. It has recently been reported that a high intake of fermented food increases microbial diversity in the gut and decreases inflammatory burden [33]. Increasing fermented food consumption may be an opportunity to prevent the burden of lifestyle diseases. A traditional Japanese diet, rich in naturally fermented food such as natto, may hold promise. Synthesis of vitamin K2 by the intestinal microflora is thought to account for the infrequent primary vitamin K deficiency in most mammalian species, including humans, as well as the observed link between vitamin K deficiency and antibiotic treatment [34,35]. However, conflicting experimental results have indicated that the synthesis of vitamin K2 by the intestinal microflora fails to counteract diet-induced vitamin K deficiency. For example, rats fed a vitamin K-deficient diet develop signs of vitamin K deficiency; however, the amounts of menaquinones in the large intestine were increased compared to a control group fed with a normal diet [36].

While more investigations are needed to fill the knowledge gap between vitamin K synthesis and bioavailability, current evidence [37] suggests that dietary vitamin K is transported by lipoproteins and taken up chemically unchanged into chylomicrons in the intestinal mucosa, secreted into the lymph system and released in the circulation. The chylomicrons then enter the liver via chylomicron remnant particles. The distribution of vitamin K forms in the liver differs from plasma, where vitamin K1 is reported to be predominantly retained in the liver. Vitamin K2, however, is distributed throughout the circulation conjugated with plasma lipids and in extra-hepatic tissues, such as bone and vasculature [38]. As the body stores relatively small amounts of vitamin K, its reserves can be quickly depleted if there is no regular supplement from the diet [38]. The vitamin K recycling system makes it possible to utilize a small amount of vitamin K to maintain its function in the γ-carboxylation of vitamin K-dependent proteins (VKDPs) and minimize the hazards of insufficient dietary vitamin K intake. From an evolutionary point of view, such a well-equipped complementary recycling system may reflect the vital role of vitamin K in human health. Vitamin K hydroquinone (VKH2) is used as a cofactor for the enzyme γ-glutamylcarboxylase (GGCX), which is essential for the activation of VKDPs, by converting the undercarboxylated Glu residue into γ-carboxyglutamic acid (Gla). This conversion is driven by the oxidation of KH2 to vitamin K-epoxide (KO). Oxidized vitamin K can be reused after reduction by vitamin K epoxide reductase (VKOR) that converts KO to vitamin K and back to VKH2, thus fulfilling a recycling process. Vitamin K antagonists (VKAs) (4-hydroxycoumarin derivatives such as warfarin) block the vitamin K cycle by inhibiting VKOR activity, which can induce the risk of drug-related vitamin K deficiency.

**Figure 2:**
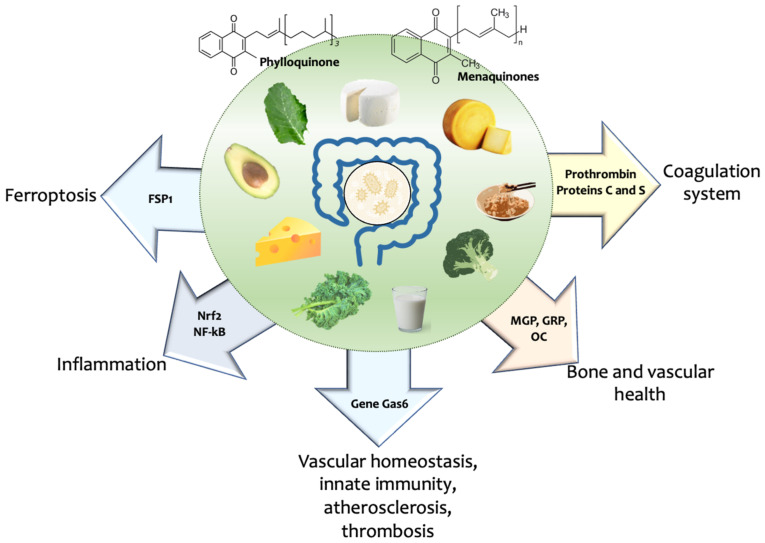
Functions of vitamin K-dependent proteins (VKDPs). Sources of vitamin K from food, or vitamin K produced by the gut microbiota, are involved in synthesising VKDPs such as prothrombin, proteins C and S related to the coagulation system. Matrix Gla protein (MGP), Gla-rich protein (GRP) and osteocalcin (OC) are extrahepatic Gla proteins involved with bone and vascular health. Vascular homeostasis, innate immunity, and atherosclerosis are associated with growth arrest-specific gene 6 (Gas6). Vitamin K may increase the expression of nuclear factor erythroid 2–related factor 2 (Nrf2) and inhibit the expression of nuclear factor kappa B (NF-kB), thus mitigating inflammation. Vitamin K may also interact with ferroptosis suppressor protein 1 (FSP1) and inhibit ferroptosis.

VKDPs are involved in various pathophysiologic pathways (Figure 2). Prothrombin (factor II), proteins C and S, are the main VKDPs of the coagulation system [39]. Extrahepatic Gla proteins, such as matrix Gla protein (MGP), Gla-rich protein (GRP) and osteocalcin (OC; BGP), play an essential role in bone and vascular health [40,41,42]. Carboxylation is also necessary for the role of VKDP growth arrest-specific gene 6 (Gas6) in vascular homeostasis, innate immunity, atherosclerosis, thrombosis, and cancer [43]. As vitamin K supplementation has a prophylactic role on vascular inflammation in type-2 diabetes by regulating the nuclear factor kappa-light-chain-enhancer of activated B cells (NF-kB)/nuclear factor erythroid 2–related factor 2 (NRF2) pathway via activation of Gla proteins [44], and that NAD(P)H quinone dehydrogenase (NQO1), one of the classic antioxidant proteins encoded by an NRF2 target gene, serves as a link between NRF2 signalling and the noncanonical vitamin K cycle [45]. The link between vitamin K and the cytoprotective transcription factor NRF2 deserves further attention. Moreover, a recent study unveiled vitamin K as the most ancient type of naturally occurring anti-ferroptotic quinone, where the ferroptosis suppressor protein 1 (FSP1)-dependent non-canonical vitamin K cycle can act to protect cells against detrimental lipid peroxidation and ferroptosis [46]. It is worth noting that aside from its enzymatic action as a cofactor of vitamin K-dependent GGCX, vitamin K2 (MK4) can target gene expression in osteoblastic cells through distinct genomic signalling, such as the pregnane X receptor-mediated (PXR) transcriptional control of the Msx2 gene [47] and phosphorylation of protein kinase A (PKA) dependent mechanisms [48].

Given the data indicating that an unhealthy diet contributes to the burden of lifestyle diseases in modern society, it is conceivable that a healthy diet is fundamental to maintaining a longer health span [26]. The significant sources of dietary vitamin K are green vegetables, fruit, dairy and cereal products, and its body stores are depleted within a few days; sufficient dietary vitamin K intake is critical for healthy living. More so, vitamin K deficiency reflects poor diet and health in general. Comprehensive reviews of the role of vitamin K in ageing and age-related dysfunctions and diseases have been summarized [11,49,50].

## 3. Vitamin K and Hallmarks of Ageing

**Figure 3:**
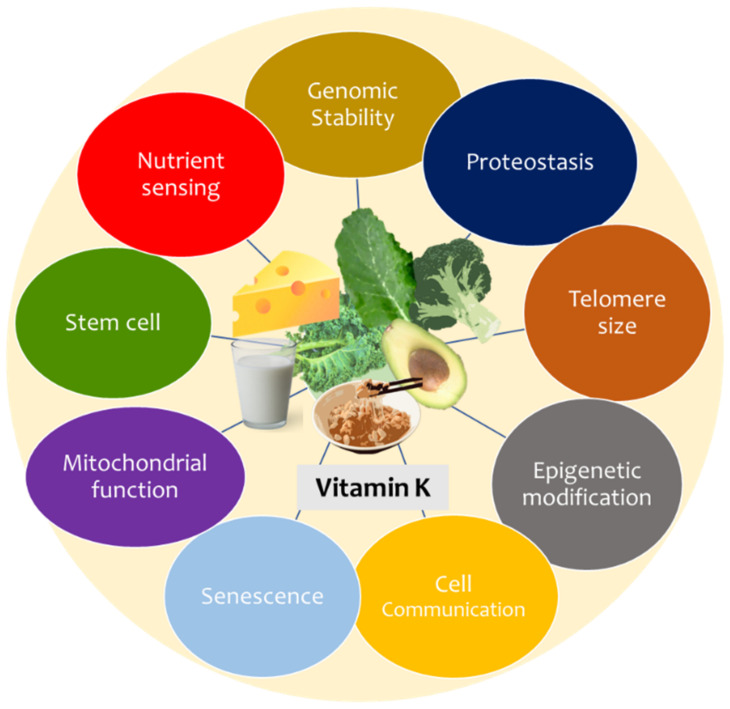
Vitamin K and the hallmarks of ageing. The hypothesis is that a possible association between vitamin K and cellular senescence exists since vitamin K plays an important antioxidant and anti-inflammatory role [54]. Additionally, it may have a role in the maintenance of the epigenome, promoting autophagy and enabling apoptosis [56].

Vitamin K has recently been attributed to antioxidant and anti-inflammatory properties [46,49]. As such, these will interplay with the hallmarks of ageing, a group of at least nine features common to ageing across taxa [51], comprising genomic instability, telomere attrition, epigenetic alterations, loss of proteostasis, deregulated nutrient-sensing, mitochondrial dysfunction, cellular senescence, stem cell exhaustion, and altered intercellular communication (Figure 3). Vitamin K can uptake reactive oxygen species (ROS) directly and thus mitigate ROS damage, a key factor driving cellular and macromolecular ageing processes [52], thus impacting hallmarks such as genomic instability, telomere attrition, cellular senescence, mitochondrial dysfunction, and epigenetic dysregulation [53]. The anti-inflammatory activity of Vitamin K also may arrest inflammageing, the chronic low-level inflammatory burden accompanying the “diseasome of ageing [27,54]. As vitamin K inhibits nuclear factor kappa B (NF-kB) activity, it may decrease inflammatory burden [52]. This may directly impact the maintenance of the methylome and, thus, ageing processes by mitigating epigenetic dysregulation [55].

Notably, while multiple existing data support the association between vitamin K deficiency and adverse health outcomes derived from observational studies, solid evidence for the salutogenic impact of vitamin K on human health remains to be fully justified. For instance, despite the established role of MGP in inhibiting vascular calcification, it remains to be elucidated whether vitamin K deficiency is directly accountable for CVD morbidity, as several randomized clinical trials (RCTs) have failed to support its cardiovascular benefits [57,58,59], particularly in high-risk populations, such as in CKD, which present with vitamin K deficiency [57,58]. A recent meta-analysis combining data from three large cohorts reported that low circulating phylloquinone concentrations were associated with an increased risk of all-cause mortality but not CVD [60]. Nevertheless, vitamin K insufficiency is detrimental to the overall health of various organs and tissues through its anti-inflammatory or anti-oxidative effect or as a cofactor for VKDPs involved in multiple pathways [11]. It is worth noting, however, that the epigenomic signatures of phylloquinone baseline status and response to supplementation may be critical players here. Recent epigenome-wide association study (EWAS) evidence has indicated that differential DNA methylation exists in several regions with previously unknown relationships to phylloquinone absorption, including the TMEM263 locus and the NPC1L1 gene [61].

### 3.1. Dietary Source of Vitamin K and Health—Food Pattern Matters

Inconsistent results from observational studies and RCTs investigating the role of vitamin K in age-related diseases have proven frustrating. We propose that the link between diet and health could provide insights to resolve these otherwise equivocal findings. For example, portion size estimation might help resolve differing data from epidemiological studies evaluating vitamin K nutrient intake through dietary recall. Moreover, a fundamental question remains regarding the dietary source and bioavailability of phylloquinone and menaquinones. For instance, green vegetables are known to be the predominant source of dietary vitamin K. It has been reported that mixed dishes [62] (e.g., meat, poultry, seafood mixed dishes; grain-based mixed dishes; Asian mixed dishes; Mexican mixed dishes; pizza; sandwiches and soups) constitute an essential weight in dietary vitamin K1 intake [21]. This suggests an underestimation in dietary vitamin K1 intake referred to by current food composition and consumption data pools. Surprisingly, apart from green vegetables, convenience food typical of the industrialized Western diet (e.g., burgers, pizza, French fries, etc.) comprises another source of phylloquinone, largely due to the content of phylloquinone-rich vegetable oils used during food preparation [21]. Hence, the observed association between phylloquinone intake and/or concentration and health outcomes can be confounded by this overlooked dietary component, as vitamin K intake may not be entirely representative of healthy eating. For instance, high K2 is derived mainly from cheese intake, which is also the main source of saturated fat intake, though studies have indicated high K2 intake can be preventive for CVD [63]. In addition, phylloquinone obtained from ready meals processed with vegetable oil (rich in phylloquinone) may have greater bioavailability than phylloquinone obtained from fresh fruits and vegetables [64]. Jones et al. [65] have shown the bioavailability of phylloquinone in the diet represented by fast food and refined cereals, with lower-than-average intakes of fruits, vegetables, and whole grains, was twice as high as meal patterns with higher-than-average intakes of fruits, vegetables, whole grains, fish, and dairy. In this regard, studies evaluating vitamin K intake through food queries (primarily represented by green vegetables) or vitamin K status (measuring circulating phylloquinone concentrations or markers of VKDPs) can be biased if such a distinction between dietary patterns is not noted. Indeed, Harshman et al. [21] have reported that although phylloquinone intake has not changed among most US adults, the distribution of foods contributing to total intake is shifting towards the current eating patterns characterized by processed food. Therefore, characterizing dietary patterns of phylloquinone intake and identifying unacknowledged food sources of vitamin K may clarify its true role in ageing and age-related physiological dysfunction. Consequently, it provides evidence for healthy eating recommendations for the general population and high-risk populations, especially those receiving VKA treatment or CKD patients.

The same issue Is pertinent to the qualification of dietary intake of menaquinones. Adequate intake of vitamin K has been limited to plant-based estimation of phylloquinone intake in the general population, partially due to the lack of standardized food composition data for menaquinones. Dietary menaquinone intake has been concentrated mainly in fermented food, resulting in a discrepancy related to food culture. For instance, in Western countries, the main source of menaquinones comes from consuming dairy products (e.g., yoghurt and cheese), while in Japan, it comes from food such as natto. Intriguingly, fermented food intake has been recently highlighted as an effective strategy to promote healthy eating in the current industrialized era, as modern diets are typically depleted in fermented foods [33]. It is worth considering that traditional fermented foods, such as natto, should be recommended as part of a healthy (salutogenic) diet. Aside from the fermented food source, Fu et al. [66] have reported that, although low in phylloquinone, processed and fresh-cut pork products from the U.S. food supply contained menaquinone-4, menaquinone-10, and menaquinone-11, and the total menaquinone contents of processed pork products were correlated with fat content. This suggests that processed and fresh-cut pork products are another rich dietary source of menaquinones that have yet to be included in dietary vitamin K intake determinations. Indeed, the US dietary recommendations do not include menaquinone intake, as the United States Department of Agriculture (USDA) Nutrient Database [67] details only the phylloquinone content of foods and current knowledge about the identification and quantification of dietary sources of menaquinones is still in its infancy. Similarly, the European Food Safety Authority (EFSA) suggests that until more is known about dietary menaquinone content and relative bioavailability, it is premature to set a separate dietary recommendation for menaquinone intake [37].

These considerations suggest that health outcomes based upon an intake of vitamin K calculated using phylloquinone intake are largely biased. This may be redressed by including other food sources in such determinations, namely processed food and red meat, despite their otherwise detrimental effects on health. Recent data from a prospective follow-up study investigating the association of meat intake with the incidence of diseases found that higher consumption of separate or combined unprocessed red and processed meat was associated with an increased risk of ischemic heart disease, pneumonia, diverticular disease, colon polyps and diabetes [68]. Moreover, a randomized controlled trial has shown that 14 days of consumption of ultra-processed vs. unprocessed food caused weight gain [14]. Therefore, an improved focus on dietary food patterns, instead of solely on total dietary vitamin K intake, could avoid generating equivocal findings and clarify the impact of vitamin K on human health. Studies on the absorption of phylloquinone in healthy adults have also shown equivoques [65,69]. To date, no definite estimation and relative determination have been made for the average absorption and bioavailability of dietary phylloquinone and menaquinones [70]). The dietary pattern profile for vitamin K, its source of synthesis, and its bioavailability requires to be established before any dietary recommendations can be placed to incorporate vitamin K in healthy eating strategies and improve health outcomes.

### 3.2. Vitamin K and Health Outcome—Nutrient vs. Food Intake

The benefit of dietary vitamin K intake (especially the menaquinones) has been demonstrated in large population-based studies, where both the Rotterdam Study and Prospect-EPIC cohort study have shown a high intake of dietary menaquinones was related to a reduction in cardiovascular events [63,71]. However, scientific evidence regarding the causal effect of vitamin K intake on health is not well-supported by RCTs. One way to improve our understanding of the null findings from some clinical trials investigating vitamin K and health (particularly cardiovascular outcomes) lies in the fact that most of the clinical trials have assessed vitamin K supplement (K1 or K2) at a specific dose, rather than quantifications of vitamin K intake from food. This resonates with several epidemiological research findings suggesting the controversial concept of nutrient supplementation and health benefits. The NHANES (National Health and Nutrition Examination Survey), involving 30,899 US adults, suggested that adequate vitamin A, vitamin K, magnesium, zinc, and copper were associated with lower all-cause and CVD mortality. The associations were confined to nutrient intake from foods but not supplements per se [72]. Similar findings were also derived from NHS (Nurs’s’ Health Study), where long-term multivitamin use was not associated with a reduced mortality risk or incident stroke [73].

Moreover, results from the systemic analysis do not support the beneficial effect of nutritional supplementation for the primary prevention of CVD or cancer [74,75]. Such nutritional research data suggest the fault of focusing on a specific nutrient instead of dietary patterns in relation to health, and the null findings of interventional trials on vitamin K and cardiovascular outcomes might be another “usual suspect” in this scenario. It is unlikely that one single nutrient (or super-nutrient) could ever be the “elixir for all” and that food intake matters more than a singular bioactive compound to ensure good health. Exploring the beneficial effects of whole foods containing certain dietary patterns and nutrients (including vitamin K) may be the most appropriate approach for future investigation regarding diet, nutrition, and health. More importantly, such findings can be directly translated into dietary recommendations for public health. Additionally, given the role of the gut microbiome in contributing to vitamin K synthesis, the impact of the contribution of different microbial populations is worth considering. This is pertinent to a recent general population study indicating differences in microbiome composition between individuals at different social-economic positions, biological age, and inflammatory status [76].

As VKDPs (e.g., MGP, GRP and osteocalcin) play an important role in skeletal and vascular health [50], it is important to know to what extent different vitamin K quinones (phylloquinone or menaquinones) can affect carboxylation of VKDPs. In the case of MGP, as one of the most extensively investigated extra-hepatic VKDPs, a heterogeneous study design with different doses of vitamin K supplements makes it difficult to compare and evaluate the change of plasma (or even tissue) dp-ucMGP levels in response to phylloquinone and menaquinones. Such questions need to be addressed as this may provide another explanation of the inconclusive findings of vitamin K supplements and health outcomes.

## 4. Vitamin K, Gut Microbiome, and Ageing

Though not fully deciphered, emerging evidence indicates that the human gut microbiome has evolved from an ancient paleo-microbiome and becoming profoundly altered in the current industrialized era [77]. Aside from factors such as modern sedentary lifestyles and medical practices, a critical partner in the crime of such a fundamental shift in gut microbiome lies in the switch of food patterns from a primitive plant-based diet toward a westernized industrialized diet characterized by excessive calorie intake, and processed food with high fat and low fibre content [78,79]. Such an evolution of the gut microbiome in response to a rapidly changing anthropogenic environment has induced significant public health concerns, where studies have shown that ‘industrialized’ lifestyles are correlated with both a lower diversity in the gut microbiome and increased incidence of the chronic burden of lifestyle diseases [78]. We have recently reported that socioeconomic position links differences in circulatory microbiota with biological age [76]. It has also been recently verified that the dynamics of the gut microbiome and its impact on metabolic pathways may not only reflect but contribute to health span and lifespan [9]. Given the pivotal role of diet in shaping the gut microbiome, a comprehensive depiction of the interplay between the gut microbiome and its metabolomes can provide insights into the complexities of the diet-microbiome-vitamin K-health diagram.

Compared to macronutrients (i.e., carbohydrates, fat, and protein), less is known about the impact of micronutrients on gut microbiome composition and metabolic profiles. Nevertheless, there is evidence that micronutrients, including vitamins (e.g., vitamin A, B12, D, E, K2) and minerals (e.g., iron, magnesium, selenium, and zinc), can influence gut microbiota composition and metabolism [80]. Specific vitamins and vitamin-derived substances synthesized by the gut microflora serve as co-growth factors for the gut bacteria [80]. This is exemplified by gut-derived vitamin B biosynthesis shared with the gut microbiota and the gut microflora [81]. Thus, micronutrient-microbiota interactions may affect human health by modulating gut microbiota composition and metabolic profiles. In this regard, vitamin K might be a modulator of diet-gut microbiota interactions that has been underestimated. As previously mentioned, while vitamin K1 (phylloquinone) is a dietary source of vitamin K, menaquinones can be obtained from the diet and gut microbiome biosynthesis. Some bacterial taxa have lost critical genes for menaquinone biosynthesis but still acquire quinone-dependent terminal reductases, suggesting a potential gut-derived conversion of phylloquinone to menaquinones [82]. Indeed, menaquinones synthesized from gut bacteria were previously identified as a necessary growth factor of other neighbouring bacteria [73]. Hence, human gut microflora depleted with genes for menaquinones biosynthesis can still utilize dietary vitamin K quinones or precursors secreted from neighbouring bacteria. In this regard, a better understanding of how the host gut can use dietary vitamin K quinones or precursors secreted from neighbouring bacteria is required.

**Figure 4:**
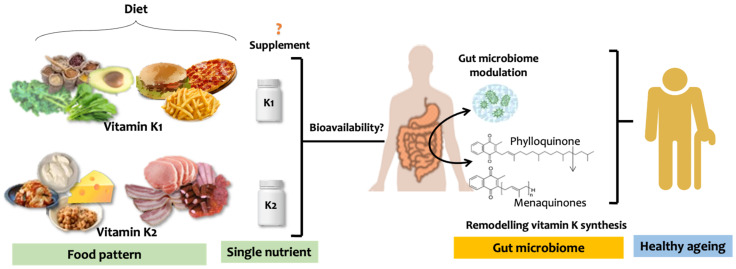
Vitamin K and healthy ageing: focus on diet-gut microbiome-health axis. Dietary sources of vitamin K, considering specific food patterns and their bioavailability, need to be addressed in estimating total vitamin K intake. The two primary forms of dietary vitamin K include vitamin K1 (phylloquinone) and vitamin K2 (menaquinones; MKn), which can be obtained from food intake or single nutrient supplements. The benefits of vitamin K1 and K2 supplements have not been fully validated, suggesting one single bioactive component may not be a silver bullet for all maladies. Implementing a healthy diet rich in vitamin K is more strategic in improving human health. The relation between dietary vitamin K and the gut microbiome is bi-directional. Vitamin K might induce nutrient-microbiota interactions and modulates gut microbiome composition characterized by favourable metabolic profiles. Furthermore, dietary vitamin K quinones can be remodelled by the gut microbiome into other menaquinones that further participate in physiological pathways.

Recent work from Eills et al. [83] has shown that caecal microbial community composition significantly differed in vitamin K-deficient female mice compared to females on vitamin K-sufficient diets (phylloquinone, MK4 and/or MK9). Moreover, using isotope-labelled vitamin K supplements, the authors found that dietary vitamin K quinones (phylloquinone, MK4 and/or MK9) were remodelled to other MKs in vivo. These findings thus provide new evidence that dietary vitamin K intake influences microbial community composition and that vitamin K quinones can be remodelled by the gut microbiome (Figure 4). Regarding the bioavailability of vitamin K, Vitamin K1 can be found in leafy vegetables (collards, spinach, kale, or broccoli), avocado, and cereal products seen as healthy. Still, also, we can find vitamin K1 in food seen as unhealthy such as burgers, pizza, and French fries, due to the content of phylloquinone-rich vegetable oils used during food preparation [21].

Similarly, vitamin K2 intake from fermented food, including yoghurt, cheese, and natto, constitutes a healthy food pattern. In contrast, processed and fresh meat products are other rich dietary menaquinones yet to be included in dietary vitamin K determinations. The total vitamin K intake may remain unchanged with a reduced healthy food intake and compensated by an elevated unhealthy food source. As such, food pattern is crucial in evaluating vitamin K intake and its impact on human health.

As vitamin K inadequacy is usually triggered by depletion of dietary vitamin K intake, the majority of vitamin K requirement in humans comes from the diet. While the full picture of dietary vitamin K and its influence on VKDPs and human health remains to be elucidated, it is plausible that bacterial synthesis of menaquinones contributes indirectly to human health through modulation of the composition of the gut microbiota, supporting the notion of nutrient-microbiota interactions. Similarly, Karl et al. [84] have reported that in humans, the interindividual variability in faecal menaquinone content was associated with variability in gut microbiota composition. Thus diet-mediated alterations in gut menaquinone content may facilitate the modulation of gut microbiome composition. Given that some gut-derived menaquinones, not abundantly available in the diet, are present in human hepatic tissue [85], it implies that at least some gut bacteria-derived menaquinones are bioactive and can be absorbed to exert physiological function. Other residual questions remain to be answered, including to what extent menaquinones in stools reflect the quantity and forms of bacterial-derived menaquinones with bioavailability and which dietary patterns contain vitamin K or vitamin K supplements (dose, intervention time) can be remodelled to bacterial menaquinones in the human gut.

Despite the emerging clues of vitamin K and gut microbiome interactions, more is needed about the direct impact of such interaction on measures of human health. A large body of research has demonstrated the role of vitamin K and VKDPs in ageing and age-related diseases [11,49,50], but these studies have seldom examined the influence of diet and/or the gut microbiome on such associations. Therefore, the diet-microbiome interaction needs to be highlighted and incorporated into future investigations on the clinical impact of vitamin K on human health. This, in turn, may elucidate some of the conflicting results from clinical trials regarding vitamin K supplements and health outcomes, where the gut microbiome profile has often been neglected.

## 5. Conclusions

Investigations into vitamin K and its role in human ageing and age-related dysfunctions are expanding and progressing exponentially. However, several critical caveats need to be acknowledged to avoid equivocal findings. Firstly, comprehensive identification of various dietary sources of vitamin K and their bioavailability requires description, among which specific food patterns need to be addressed as possible confounders in estimating total vitamin K intake. As the Westernized diet has shifted from fresh vegetables, fruits, and whole grain intake into an urbanized diet (i.e., less fibre, more meat, and processed food products), the total vitamin K intake may remain unchanged, with a reduced vegetable intake compensated by an elevated meat/processed food source [21] (Figure 4). This can undeniably challenge the notion of sufficient vitamin K intake representing a healthy diet. As such, food pattern is crucial in determining vitamin K source and its impact on human health (i.e., quality matters more than quantity). Secondly, a thorough consideration of the interplay between diet-microbiome and health needs to be implemented to evaluate vitamin K’s impact on human health. Emerging evidence supports the role of vitamin K as a modulator of gut microbiome composition. Changes in microbial composition concerning vitamin K/dietary pattern and its direct impact on clinical health outcomes have yet to be addressed. Well-designed future studies are warranted to reveal the complexity of such diet-microbiome interactions mediating vitamin K and health outcomes. Thirdly, aside from the fact that vitamin K has the potential to act as a super nutrient providing various health benefits, it is worth noting that one single bioactive component is not a panacea for all maladies. Lessons can also be learned from other such scenarios; for example, the use of flavonoids, widely present in various plant foods, are believed to influence gut microbiota and host health. However, results from an interventional study designed to evaluate the effect of fruit and vegetable flavonoids on the gut microbiota have shown that changes in selected gut bacteria were not triggered by the flavonoid content. The authors concluded that such effects were likely to be modified by other bioactive components [86]. This echoes the concept of food patterns and health, where exploring the beneficial effects of whole foods and the food groups that compose dietary patterns and vitamin K may be more strategic for future exploration of the microbiome and health. As such, evidence of vitamin K-related healthy eating patterns can be directly implemented into dietary recommendations for public health. Fourthly, it must be noted that due to the high interindividual variability in eating habits and microbiome profiles, an optimal and balanced diet-microbiome-health pattern has not been identified. Future research incorporating vitamin K into the diet-microbiome-health axis should evaluate microbiota-mediated metabolites, an important link to address the impact of microbiome profile on human health. Some ideal microbiome compositions and metabolic activities may exist, which might be unique for each person. While this may complicate the interpretation and generalization of research findings, it can alternatively provide a certain rationale for a personalized nutrition-health model that can maximize its public health effect.

## Data Availability

Not applicable.
